# Why are not all paediatric cancer patients treated with protons? A population-based report from Sweden, 2016–2023

**DOI:** 10.2340/1651-226X.2025.43726

**Published:** 2025-09-02

**Authors:** Anna Asklid, Ingrid Kristensen, Ulla Martinsson, Martin P. Nilsson, Malin Blomstrand, Måns Agrup, Anna Flejmer, Anna-Maja Svärd, Charlotta Fröjd, Erik Almhagen, Jacob Engellau, Anna Embring

**Affiliations:** a Department of Oncology, Karolinska University Hospital, Stockholm, Sweden; b Department of Oncology-Pathology, Karolinska Institute, Stockholm, Sweden; c Department of Hematology, Oncology and Radiation Physics, Skane University Hospital, Lund, Sweden; d Department of Oncology, Clinical Sciences, Lund University, Lund, Sweden; e Department of Immunology, Genetics and Pathology, Uppsala University, Uppsala, Sweden; f Department of Oncology, Sahlgrenska University Hospital, Gothenburg, Sweden; g Department of Oncology, Institute of Clinical Sciences, University of Gothenburg, Sweden; h Department of Oncology, and Department of Biomedical and Clinical Sciences, Linköping University, Linköping, Sweden; i Department of Radiation Sciences, Oncology, Umeå University, Sweden; j Department of Nuclear Medicine and Medical Physics, Karolinska University Hospital, Stockholm, Sweden

**Keywords:** Radiotherapy, paediatrics, proton therapy, clinical practice pattern

## Abstract

**Background:**

In 2015, a proton therapy (PT) facility was established in Sweden with one aim being to ensure access for all children expected to benefit from PT. Despite potential dosimetric advantages and full subsidisation, PT is not always selected. This study explores reasons for choosing alternative radiotherapy (RT) modalities in a paediatric population.

**Material and methods:**

RT courses delivered to patients ≤ 18 years during 2016–2023 were identified from a national registry. Medical records were retrospectively reviewed to identify reasons for not selecting PT.

**Results:**

Only 34% (*n* = 275) of all courses identified were delivered with PT. Of the remaining 66% (*n* = 544), 90% were photon RT, 9% combined PT and photon RT, and 1% electron RT. Among photon RT courses, 97% were delivered with conventional external beam radiotherapy (EBRT), 2% with stereotactic radiotherapy (SRT), and 1% with brachytherapy. The most common reason for choosing photons was non-curative intent (35%), followed by equal or superior expected outcome compared to PT (23%), total body irradiation (TBI) (15%), and uncertainties due to air, organ motion, or metal in field (15%). Dosimetric comparison led to the selection of a favourable or equal photon plan in 8%. Logistical, social, and technical reasons constituted 4%.

**Conclusion:**

While PT can reduce radiation exposure to healthy tissues, particularly important in children, clinical, logistical, and technical factors often necessitate alternative RT modalities. This study highlights the importance of individualised RT planning and multidisciplinary collaboration to balance medical, technical, and practical considerations to ensure optimal treatment approach in every child.

## Introduction

Over past decades, the use of radiotherapy (RT) has declined but remains a cornerstone within the multimodal treatment concept for paediatric malignancies [1]. Various RT techniques are applied across a broad range of paediatric diseases, predominantly for malignant and occasionally benign conditions [2–4]. In Sweden, around 350 children are diagnosed with cancer annually. About 25% of these will receive radiation as part of their treatment [5].

Modern conformal RT techniques have continuously evolved over time and proton therapy (PT) represents a major advancement in cancer treatment offering dosimetric advantages by minimising radiation exposure to healthy tissues [6]. Although long-term clinical data remains limited, PT is widely recognised as standard of care for many paediatric malignancies, given its potential to lower the risk of severe late side effects and secondary malignancies [7–9].

In late 2015, a national proton facility opened in Uppsala, Sweden, with one aim being to provide treatment to paediatric patients expected to benefit from PT [10, 11]. At this time, PT use in paediatric cancer showed considerable international variation. Central nervous system (CNS) tumours were the most common diagnoses treated with PT worldwide. However, only a limited number of patients were treated with PT in each centre and a wide range of diagnoses were reported [12]. PT was available in Sweden before 2015, at The Svedberg laboratory (TSL), but with treatment in sitting position and hence used mainly for CNS and head/neck targets and without possibilities to treat patients in need of anaesthesia [11, 13].

All paediatric cancer patients in Sweden are discussed at multidisciplinary tumour boards and all paediatric RT cases are reviewed within the Swedish Working Group of Paediatric Radiotherapy (SBRTG), comprising radiation oncologists working in close collaboration with dosimetrists and physicists at the six paediatric RT centres. The standard approach is to generate comparative photon and proton plans. These are assessed at SBRTG’s bi-weekly national video conferences, where the most appropriate plan is selected for each individual patient. A back-up photon plan is always available for patients receiving PT (except for craniospinal irradiation, CSI), to avoid prolonged treatment interruptions caused by unforeseen technical issues.

Treatment of paediatric cancer, including travel costs and accommodation, is fully subsidised in Sweden and entails no additional financial issues for the families compared to other RT options. Since the opening of the proton facility, experiences have been positive with few serious side effects [13]. The aim of this study was to investigate reasons why paediatric patients in Sweden were treated with an RT modality other than PT, despite PT being readily available.

## Material and methods

This observational retrospective cohort study includes all paediatric patients treated with an RT modality other than PT in Sweden during 2016–2023. Treatment courses, clinical and treatment characteristics were identified through RADTOX, a prospectively maintained national RT database [13]. Courses were grouped by treatment year, centre, modality, and diagnosis. Courses including an RT modality other than solely PT were identified. Radiation oncologists from the six paediatric RT centres in Sweden retrospectively reviewed patient records to determine reasons for not selecting PT. In cases where photon therapy was chosen, it was documented whether comparative photon and proton treatment plans had been made.

## Statistical analysis

Clinical and treatment characteristics are presented using descriptive statistics. Chi-squared statistics assessed treatment distribution of PT versus non-PT treatment across centres. The Kaplan–Meier estimator was used to estimate overall survival (OS), calculated from the start of the first RT until death or closure of database (31 December 2023), whichever came first. Analyses of OS were performed using R Statistical Software (4.3.3; R Core Team 2024) [14] using ggplot [15] and ggsurvfit packages [16].

## Ethics declarations

The study was performed in accordance with current ethical principles and approved by the National Ethical Review Authority (Dnr 2023-04736-02).

## Results

In total, 819 RT courses were identified in the RADTOX registry during 2016–2023. Of these, 275 (34%) were delivered with PT to 263 individual patients (57% male, 43% female) and 544 (66%) with other modalities (non-PT), to 464 patients (54% male, 46% female). Twenty-five patients received both PT and non-PT at different timepoints during the study period.

**Figure 1:**
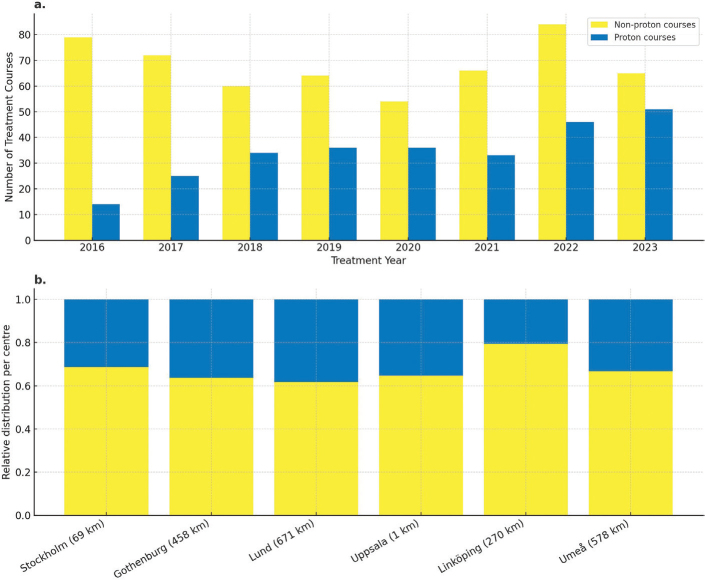
Annual distribution of radiotherapy courses delivered using proton therapy (blue) and non-proton therapy (yellow) between 2016 and 2023 (A). Relative distribution of treatment courses per paediatric radiotherapy centre, with distance to the national proton facility in kilometers (km) indicated in brackets (B). There was no significant association between proton therapy use and the distance from the proton facility (Chi-squared test, p = 0.2).

Non-PT remained predominant but declined over time (Figure 1A). PT comprised 40–45% of annual courses, peaking in 2023 after a dip in 2021. PT use was relatively consistent across centres and not significantly associated (*p* = 0.2) with geographic distance from the proton facility (Figure 1B).

**Figure 2:**
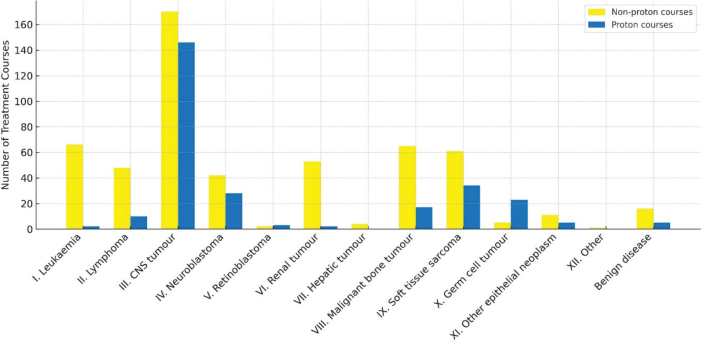
Distribution of diagnosis groups for all proton (blue) and non-proton (yellow) treatment courses over the study period. CNS: central nervous system.

Diagnoses were classified using International Classification of Childhood Cancer (ICCC-3) [17]. CNS tumours dominated, accounting for one-third of non-PT and over half of PT courses (Figure 2). Other common diagnoses in both groups were sarcoma and neuroblastoma even though a peak in PT for neuroblastomas was noted at the end of the study period. Leukaemias and renal tumours were mainly treated with non-PT. See supplementary material, Table S1 and Figure S1 for details.

## Non-proton cohort

**Figure 3:**
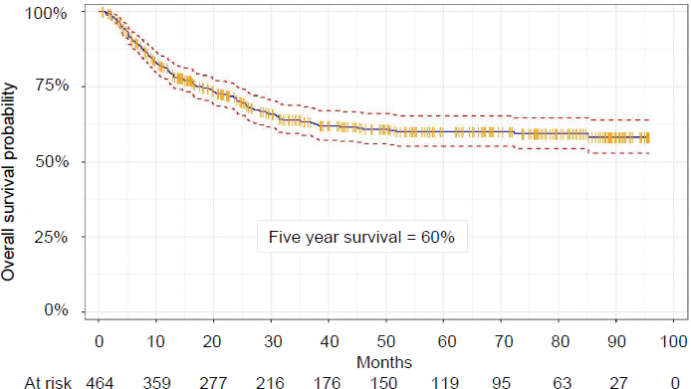
Kaplan–Meier estimate of overall survival in the non-proton group (n = 464). Solid blue curve is the Kaplan–Meier estimator. Red dashed curves show the 95% confidence interval. Orange vertical markers show censored patients. n: number of patients.

The non-PT group (*n* = 544) constituted the primary study cohort for analysis. Median age at start of first RT was 9.6 years (range 0.1–18.8 years). After a median follow-up time of 26.6 months (range 0.6–96 months), the median OS was not reached, and the 5-year OS was 60% (Figure 3). Most patients (87%) received one RT course while the remainder had two to seven courses during the study period.

Photon RT was used in 90%, combined proton-photon treatment during the same course in 9%, and electron RT in 1%. Of photon RT courses, 97% were external beam radiotherapy (EBRT) (two-dimensional [2D], three-dimensional conformal radiotherapy [3D-CRT], and rotational techniques), 2% stereotactic radiotherapy (SRT), and 1% brachytherapy. SRT included 10 courses in lung and CNS lesions, of which six were delivered with Gamma Knife. Brachytherapy was registered in seven localised rhabdomyosarcomas, five in the urogenital tract, and two in the nose. Electron RT was used in three superficial skin lesions.

**Figure 4:**
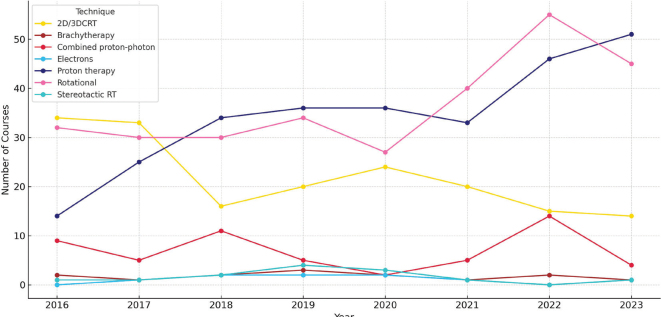
Trends in the use of non-proton radiotherapy techniques and proton therapy over the study period. 2D: two-dimentional; 3D: three dimentional; CRT: conformal radiation therapy; RT: radiotherapy.

A shift in RT techniques occurred over time, with declining use of 2D/3D-CRT and increasing use of rotational techniques (Intensity modulated radiation therapy (IMRT), volumetric modulated arc therapy (VMAT), and helical tomotherapy), becoming the most applied photon technique from 2020. By 2023, rotational techniques accounted for over two-thirds of all non-proton RT courses (Figure 4). Similarly, in the subgroup of total body irradiation (TBI), total marrow irradiation (TMI), or total lymphoid irradiation (TLI), a notable shift to rotational techniques was observed, accounting for 64% in the last 2 years of the study period (data not shown).

## Rational for photon RT

The most reported reasons for choosing EBRT were non-curative intent (35%), equal or superior expected outcome (clinical and/or dosimetrical) (23%), and TBI/TMI/TLI (15%) (Table 1). Other reasons included uncertainties with PT due to tissue heterogeneities (e.g. air-bone interface), organ motion or metal in field (15%), dosimetrically favourable (5%) or equal (3%) photon plan, and logistical (2%), social (1%), and technical factors (1%).

**Table 1:** Reasons for choosing conventional external beam radiotherapy (n = 473).

Reason	Number of courses (%)	Comparative proton-photon plans (%)
Non-curative intent	167 (35)	0 (0)
Equal or superior expected outcome	109 (23)	0 (0)
TBI/TMI/TLI	73 (15)	N/A
Uncertainty due to air/movement/metal	71 (15)	27 (38)
Favourable photon plan	22 (5)	22 (100)
Equal photon plan	13 (3)	13 (100)
Need for urgent treatment start (logistical)	9 (2)	4 (44)
Social considerations	6 (1)	0 (0)
Technical reasons	3 (1)	0 (0
**Total**	**473**	**66 (14)**

n: number of courses; TBI: Total body irradiation; TMI: Total marrow irradiation; TLI: Total lymphoid irradiation; N/A: Not applicable.

**Figure 5:**
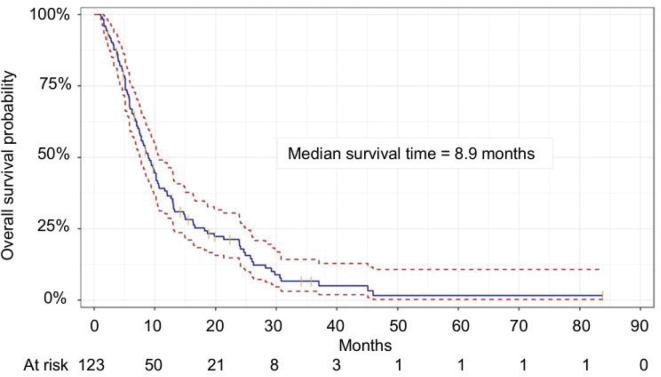
Kaplan–Meier estimate of overall survival in the non-curative intent group (n = 123). Solid blue curve is the Kaplan–Meier estimator. Red dashed curves show the 95% confidence interval. Orange vertical markers show censored patients. n: number of patients.

In the non-curative group (*n* = 167), where treatment aimed to relieve symptoms, achieve local control, or prolong life when cure was not deemed achievable, CNS tumours (68%) particularly diffuse intrinsic pontine glioma (DIPG) and diffuse midline glioma (DMG) dominated (Table 2). After a median follow-up of 8.4 months (range 1.1–83.9 months), the median OS was 8.9 months (Figure 5). Four patients died within 30 days of starting RT. See supplementary material, Table S2 for details on fractionation.

**Table 2:** Distribution of diagnoses in the non-curative intent group (n = 167).

Diagnosis group (*n*/%)	Diagnosis subgroup	Number of courses (%)
CNS tumour (114/68)	DIPG/DMG	65 (39)
	Other high-grade glioma	29 (17)
	Medulloblastoma	9 (5)
	Ependymoma	4 (2)
	Low-grade glioma	3 (2)
	Other high-grade CNS tumour	2 (1)
	Intracranial embryonal tumour	1 (1)
	Meningioma	1 (1)
Malignant bone tumour (31/19)	Ewing sarcoma	16 (10)
	Osteosarcoma	13 (8)
	Other malignant bone tumour	2 (1)
Soft tissue sarcoma (10/6)	Rhabdomyosarcoma	7 (4)
	Other soft tissue sarcoma	3 (2)
Neuroblastoma (6/4)	Neuroblastoma	6 (4)
Hepatic tumour (4/2)	Unspecified hepatic tumour	4 (2)
Other and unspecified malignant neoplasm (1/1)	Pleuropulmonary blastoma	1(1)
Other malignant epithelial neoplasm (1/1)	Nasopharyngeal carcinoma	1(1)
**Total**		**167 (100)**

n: number of courses; CNS: Central nervous system; DIPG: Diffuse intrinsic pontine glioma; DMG: Diffuse midline glioma.

In 86% of EBRT courses, including all with reasons stated as non-curative intent, equal or superior expected outcome, TBI/TMI/TLI (considered not applicable) social and technical circumstances, no comparative proton–photon planning was performed (Table 1). For the remaining 14%, comparative planning was conducted, but PT was not selected for various reasons described further in the text. Use of comparative planning generally increased over time.

The second largest group, equal or superior expected outcome (*n* = 109), mainly included Wilms tumour or neuroblastoma with large-volume targets (e.g. whole abdomen, lung, flank) and low prescribed doses (10–25.2 Gy) (See supplementary material, Table S3). In 13 cases, comparative plans showed equivalency, typically in similar diagnoses and targets. Additionally, in some sarcomas located distally or superficially in extremities, photon plans offered no clear disadvantage. In some of these cases, additional social and practical considerations further contributed to selecting photon RT over PT.

Photon RT was preferred in 71 cases where uncertainties related to metal in field (e.g. bone sarcomas), tissue inhomogeneities, or internal organ movement (e.g. head and neck area, thorax, abdomen) posed challenges with PT (see supplementary material, Table S4). In another 22 cases, photon plans were favoured due to factors like better target coverage, robustness or reduced uncertainties associated with the wider proton penumbra near sensitive organs at risk (OARs), most commonly in targets with a large interface against the brainstem and high prescribed doses up to 59.4 Gy (see supplementary material, Table S5). Photon plans were also preferred for small orbital targets, to avoid hotspots more frequently observed in PT plans, and in one ocular case the photon plan achieved better coverage with bolus. Concerns about increased biological effect due to elevated linear energy transfer (LET) in the brainstem influenced decisions in a patient with central respiratory impairment. In another patient, type 1 reirradiation (i.e. a new course of RT with geometrical overlaps with a previously irradiated volume) in a region earlier treated with Gamma Knife also affected the clinical decision. Furthermore, in one extremity sarcoma, the photon plan offered better sparing of the limb circumference using tangential fields.

Social factors (*n* = 6) related to school/family context influenced decisions, particularly in centres far from the PT facility. Two of these cases involved CSI delivered with helical tomotherapy to minimise normal tissue exposure. Urgent symptoms (*n* = 9) further prompted initiation of RT at the home clinic faster than would have been possible with PT. Technical limitations (*n* = 3) included treatment volumes exceeding the capacity of the PT planning system.

## Combined proton–photon courses

In 9% of courses, protons and photons were combined in the same course. One-third was mixed due to unplanned technical downtime at the proton facility, where at least one fraction was delivered with photons to avoid prolonged treatment interruption. Another third involved acute symptoms (mainly CNS-related) requiring urgent treatment start, before a start with PT could possibly be arranged. In the final third, a combined approach was deemed dosimetrically advantageous, mostly in whole lung RT cases treated with photons followed by a proton boost exploiting the strengths of both modalities (see supplementary material, Table S6). In some CNS-cases, a mixed plan was chosen as photons offered superior target coverage, while protons reduced the integral dose and OAR exposure. Finally, four patients treated in early 2016 still received CSI with photons but also the boost with protons prior to full implementation of PT-based CSI.

## Discussion

This nationwide registry-based study provides a comprehensive overview of paediatric RT practice in Sweden from 2016 to 2023, offering real-world insights into clinical decision-making. Nationally, PT use has steadily increased, reaching 40–45% of all paediatric RT courses. However, this is still lower than the 60–80% initially projected before the centre opened [18]. The temporary dip in PT use observed in 2020 was likely due to the Covid-19 outbreak. A relatively even distribution of PT use across centres, regardless of distance from the proton facility, suggests that treatment decisions are primarily guided by clinical and dosimetric considerations, not geographic proximity.

The most common reason for selecting a non-PT approach was non-curative intent, reflecting prioritisation of symptom relief and quality of life over potential long-term benefits in patients with limited life expectancy. Palliative RT remains a valuable tool to alleviate or prevent symptoms in paediatric patients [3, 19]. Careful patient selection is essential to balance potential benefits against disadvantages such as toxicity or additional time spent in hospital near the end of life. While this study did not aim to assess treatment outcomes, the low 30-day mortality (3%) suggests appropriate patient selection, consistent with findings from other paediatric-focused studies but considerably lower than in the adult population [3, 20]. Social circumstances also influenced decision-making in a small subset of cases, emphasising the need for patient-centred, individualised care.

Several reasons for selecting non-PT treatment were described even in curative settings, despite the availability and full subsidisation of PT. While clinical outcomes with PT are encouraging and its use is expected to increase [21], other RT modalities will remain necessary. Photon RT for instance, continues to be standard of care for TBI [22], and in line with international guidelines we also observed a trend towards increased use of rotational techniques such as VMAT and helical tomotherapy in our TBI treatments over the years [22, 23].

Another frequent rationale for selecting photon RT was the anticipated equivalence in clinical outcome of photon and proton treatment without comparative planning being performed. This was particularly noted in treatments involving large target volumes, such as whole lung, whole abdomen, or flank irradiation in Wilms tumours or neuroblastomas. One explanation may be the relatively low prescribed doses (10–25.2 Gy) and the fact that photon RT has been the standard treatment in many historical paediatric study protocols. However, this represents an area for potential improvement, as PT can reduce low-dose exposure and spare OARs, thereby mitigating acute toxicity and potentially long-term effects even at lower radiation doses [24, 25]. Notably, the use of PT for neuroblastoma increased towards the end of the study period and our recently published data on comparative proton and photon treatment planning in this patient group further support the benefits of PT in selected cases by lower doses to OARs and reduced low-dose exposure to surrounding healthy tissue [26].

Mixed proton–photon treatments were used to a lesser extent, reflecting a hybrid approach, with potential to combine strengths of both modalities [27, 28]. In some cases, this approach was necessitated by technical downtime, urgent treatment needs, or other clinical considerations that favoured one modality for at least part of the course. This demonstrates opportunities to balance dosimetric advantages with logistical realities and further illustrates the complexity of paediatric RT.

Uncertainties related to air interfaces and internal organ motion contributed to the choice of photon therapy in a substantial number of courses. This may partly reflect limited experience with motion management techniques at the PT centre, especially in the beginning of the study period. Over time, an increasing trend in comparative dose planning was shown, probably mirroring implementation of motion management strategies (e.g. 4DCT and breath hold techniques), even for children and adolescents. Improved strategies to address technical challenges together with streamline PT logistics, and preparing children as much as possible before starting RT to reduce the need of anaesthesia, could enhance feasibility for a broader group of paediatric patients [29, 30].

Photon RT was sometimes preferred or considered equivalent to PT in terms of target coverage, robustness, and the ability to create steep dose gradients – particularly near critical structures like the brainstem, where the wider lateral penumbra of protons might pose challenges [6, 31]. In cases of type 1 reirradiation [32] and concerns of increased LET, advanced photon techniques (e.g. IMRT, VMAT) may represent a reasonable alternative [33, 34]. In our cohort, this rationale applied in selected ependymoma cases with a large interface against the brainstem, probably reflecting previously raised concerns about PT-related brainstem toxicity. Limitations in technical infrastructure, such as the absence of more advanced and accurate dose calculation algorithms and LET evaluation tools in daily clinic, may also have influenced the decision-making, especially in anatomically complex cases. While large prospective randomised trials are unlikely due to practical and ethical reasons, PT is increasingly used for these challenging targets and growing outcome data suggest no clear correlation between brainstem toxicity and RT modality [35] and PT should always be considered in multidisciplinary discussions for all children with CNS tumours [36, 37]. Reporting of outcome data is furthermore strongly encouraged, alongside continuous improvements in PT technology, access to advanced treatment planning systems to address dose-deposition uncertainties, developments that may support and aid clinical decision-making and broaden the applicability of PT to more paediatric patients.

Other techniques limiting radiation exposure to surrounding healthy tissues, such as brachytherapy and SRT, remained infrequently used throughout the study period. Their limited application likely reflects both narrow clinical indications and high demands in terms of equipment, expertise, and treatment planning [4]. Nevertheless, these techniques remain valuable in selected cases, for example localised rhabdomyosarcoma, where preserving organ and function can be very important for quality of life, offering clinicians additional opportunities to individualise care [38, 39].

RT decisions in the paediatric population require multidisciplinary discussions to weigh the benefits and limitations of each modality. Over time, a distinct increase in the utilisation of comparative proton-photon planning was observed and this upward trend may reflect growing awareness of the benefits of plan comparison to guide modality selection. Sweden’s model of distributed competence – where RT planning is conducted at six regional centres, while PT delivery is centralised – may contribute to variability in local experience [40]. In the initial years of PT use, some referring centres may have had limited hands-on experience with proton planning, possibly leading to more cautious or conservative modality choices in ambiguous cases. However, regular and close collaboration between clinicians and physicists in daily practice, along with continuous national coordination within SBRTG has facilitated shared expertise as all paediatric patients planned for RT, regardless of modality, are presented at SBRTG meetings.

Our study’s strengths lie in its comprehensive national cohort, ensuring representativeness and minimal selection bias. The inclusion of all paediatric RT patients treated with modalities other than PT over an extended period provides valuable insights into the decision-making processes. Limitations include the retrospective nature and the registry-based approach with inherent weaknesses such as potential underreporting of nuanced factors influencing treatment decisions. The absence of consistent comparative dose plans restricts the ability to fully assess the theoretical benefits of PT as well as the lack of information on side-effects. Although this was outside the scope of this study, it might add an important dimension in further studies. Our findings may also have limited generalisability to other healthcare systems due to differences in infrastructure and access to PT.

## Conclusion

This study provides a comprehensive overview of paediatric RT in clinical routine care with a particular focus on the rationale behind the decision-making for selecting alternative modalities than PT. Despite full subsidisation and availability, PT was used in only one third of all courses. Our findings indicate that while PT is gaining ground, photon RT – particularly with advanced rotational techniques and broad clinical indications – continues to play a major role in paediatric RT. Individualised treatment selection is especially critical in paediatric cases, where the balance between effective tumour control and sparing of normal tissue is paramount. This study contributes to support the difficult decision-making in a complex clinical setting and underscores the vital role of paediatric radiation oncologists in multidisciplinary teams. It also highlights the importance of continued close collaboration between physicians, physicists, and dosimetrists as well as within the broader RT community, both nationally and internationally, to ensure the most appropriate treatment for each child.

## Author contributions

AA, AE, UM, IK, JE, MN, MB, CF, AF, MA, AMS were involved in the planning of the study, data collection, and data analysis. AA, AE, UM, and IK prepared the manuscript. AA and AE were responsible for the literature research. AA, AE, and EA performed the statistical analyses. All authors reviewed, edited, and approved the final manuscript.

## Supplementary Material



## Data Availability

Raw data are available from the corresponding author on request.

## Supplementary material

See supplementary material for STROBE Statement-Checklist and for supplementary tables and figures.
