# Supplementary material for: Why are not all paediatric cancer patients treated with protons? A population-based report from Sweden, 2016–2023
Source: Acta Oncol. 2025 Sep 2;64:43726. doi: 10.2340/1651-226X.2025.43726 (PMC12416337; doi:10.2340/1651-226X.2025.43726)
Supplement: Supplementary file 1 [file AO-64-43726-s1.pdf]

Supplementary material has been published as submitted. It has not been copyedited, or typeset by Acta Oncologica

Supplementary material

Table S1. Distribution of proton (n=275) and non-proton (n=544) treatment courses by diagnosis group (ICCC-3) and subgroups of diagnoses.

Diagnosis group (ICCC-3)	Diagnosis subgroup	Non-PT courses (%)	PT courses (%)
I. Leukaemia	Acute lymphocytic leukaemias	59 (11)	2 (1)
	Acute myeloid leukaemias	3 (1)	0 (0)
	Myelodysplastic syndrome	1 (0)	0 (0)
	Unspecified and other leukaemias	3 (1)	0 (0)
II. Lymphoma	Hodgkin lymphomas	38 (7)	10 (4)
	Non-Hodgkin lymphomas	2 (0)	0 (0)
	Unspecified lymphomas	8 (1)	0 (0)
III. CNS tumour	Diffuse intrinsic pontine glioma/ Diffuse midline glioma	66 (12)	1 (0)
	Other high-grade gliomas	35 (6)	9 (3)
	Medulloblastomas	27 (5)	68 (25)
	Ependymomas	19 (3)	30 (11)
	Intracranial embryonal tumours	9 (2)	12 (4)
	Other high-grade CNS tumours	6 (1)	4 (1)
	Low-grade gliomas	5 (1)	0 (0)
	Meningiomas	2 (0)	6 (2)
	Craniopharyngiomas	1 (0)	7 (3)
	Choroid plexus tumour	0 (0)	1 (0)
	Malignant tumour of the pineal gland	0 (0)	5 (2)
	Unspecified CNS neoplasms	0 (0)	3 (1)
IV. Neuroblastoma	Neuroblastoma	42 (8)	28 (10)
V. Retinoblastoma	Retinoblastoma	2 (0)	3 (1)
VI. Renal tumour	Wilms tumour	50 (9)	2 (1)
	Unspecified renal tumours	3 (1)	0 (0)
VII. Hepatic tumour	Unspecified hepatic tumours	4 (1)	0 (0)
VIII. Malignant bone tumour	Ewing sarcomas	42 (8)	12 (4)
	Osteosarcomas	19 (3)	1 (0)
	Other malignant bone tumours	4 (1)	4 (1)
IX. Soft tissue sarcoma	Rhabdomyosarcomas	35 (6)	25 (9)
	Other specified soft tissue sarcoma	13 (2)	2 (1)
	Unspecified soft tissue sarcoma	11 (2)	5 (2)
	Infantile fibrosarcoma	1 (0)	0 (0)

	Peripheral nerve sheet tumour	1 (0)	2 (1)
X. Germ cell tumour	Intracranial germinomas	5 (1)	22 (8)
	Malignant gonadal germ cell tumour	0 (0)	1 (0)
XI. Other epithelial neoplasm	Nasopharyngeal carcinomas	7 (1)	0 (0)
	Salivary gland carcinomas	3 (1)	4 (1)
	Carcinomas of cervix uteri	1 (0)	0 (0)
	Thymoma	0 (0)	1 (0)
XII. Other	Pleuropulmonary blastoma	1 (0)	0 (0)
Benign diagnoses*	Benign haematological disease	12 (2)	0 (0)
	Paragangliomas	2 (0)	0 (0)
	Arteriovenous malformation	1 (0)	4 (1)
	Intravascular papillary endothelial hyperplasia	1 (0)	0 (0)
	Benign tumour of the hypophysis	0 (0)	1 (0)
Total		544 (100%)	275 (100%)

*Not classified according to ICCC-3

ICCC-3: International Classification of Childhood Cancer (Third edition), non-PT: non proton radiotherapy, PT: proton radiotherapy

Figure S1. Proportion of proton (blue) and non-proton (yellow) treatment courses per diagnosis group and year 2016-2023.

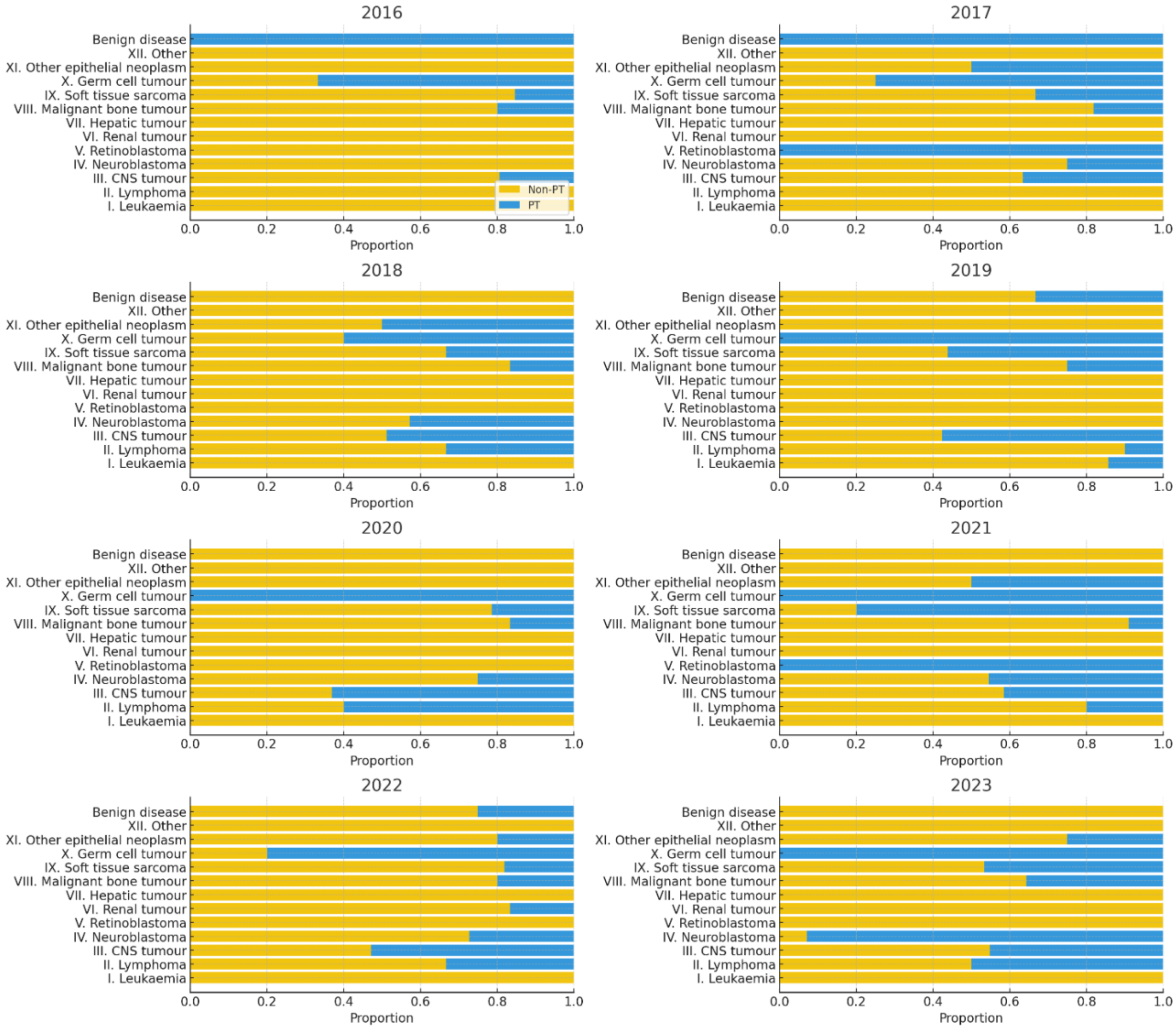

Table S2. Diagnosis and fractionation in the non-curative intent group (n=167)

**Diagnosis group**	**Diagnosis subgroup**	**Number of courses**	**Dose/fraction (Gy)**	**Number of fractions**	**Total dose (Gy)**
**CNS tumour (n=114)**	**DIPG/DMG (n=65)**				
	Primary treatment (n=39)	36	1.8	30	54
		1	3	12	36
		1	1.8	20	36
		1	1.8	19	34.5
	Relapse treatment (n=26)	12	2	10	20
		5	4	5	20
		2	1.8	11	19.8
		1	1.8	20	36
		1	3	8	24
		1	1.8 / 3	4 and 10	37.2
		1	2 / 1.8	3 and13	29.4
		1	2	7	14
		1	4	2	8
		1	1.8	2	3.6
	**Other high-grade glioma (n=29)**				
	Primary treatment (n=17)	8	1.8	30	54
		4	2	30	60
		1	1.8	33	59.4
		1	2.67	15	40.05
		1	1.8	29	52.2
		1	1.8	27	48.6
		1	1.8	22	39.6
	Relapse treatment (n=12)	5	2	10	20
		2	5	4	20
		1	2	30	60
		1	3.5	10	35
		1	3.4	10	34
		1	2	8	16
		1	1.8	11	19.8
	**Medulloblastoma (n=9)**				
	Relapse treatment (n=9)	2	1.8	30	54
		1	8	5	40
		1	2.67	15	40.05
		1	2.5	15	37.5
		1	6	5	30

		1	9	3	27
		1	1.8	12	21.6
		1	4	5	20
	**Ependymoma (n=4)**				
	Relapse treatment	2	4	5	20
		1	2	10	20
		1	1.8 /3.3	8 and 7	37.5
	**Low-grade glioma (n=3)**				
	Primary treatment (n=1)	1	3.4	10	34
	Relapse treatment (n=2)	1	1.8	30	54
		1	2	10	20
	**Other high-grade CNS tumour (n=2)**				
	Primary treatment (n=1)	1	1.8	30	54
	Relapse treatment (n=2)	1	2	10	20
	**Intracranial embryonal tumour (n=1)**				
	Primary treatment (n=1)	1	1.8	30	54
	**Meningioma (n=1)**				
	Relapse treatment (n=1)	1	4	5	20
**Malignant bone tumour (n=31)**	**Ewing sarcoma (n=16)**				
	Primary treatment (n=2)	1	1.8	36	64.8
		1	1.8	25	45
	Relapse treatment (n=14)	5	4	5	20
		3	5	4	20
		1	8	1	8
		1	3	13	39
		1	2	30	60
		1	1.8	33	59.4
		1	1.8	30	54
		1	1.8	28	50.4
	**Osteosarcoma (n=13)**				
	Primary treatment (n=2)	1	8	1	8
		1	7	3	21

	Relapse treatment (n=11)	6	4	5	20
		2	8	1	8
		1	8	2	16
		1	3.5	8	28
		1	3	10	30
	**Unspecified malignant bone tumour (n=2)**				
	Primary treatment (n=1)	1	2.5	17	42.5
	Relapse treatment (n=1)	1	4	5	20
**Soft tissue sarcoma (n=10)**	**Rhabdomyosarcoma (n=7)**				
	Primary treatment (n=1)	1	1.8	28	50.4
	Relapse treatment (n=6)	2	3	10	30
		1	4	4	16
		1	4	2	8
		1	2	25	50
		1	2	15	30
	**Other soft tissue sarcoma (n=3)**				
	Primary treatment (n=1)	1	2	18	36
	Relapse treatment (n=2)	1	5	4	20
		1	4	5	20
**Neuroblastoma (n=6)**	**Neuroblastoma (n=6)**				
	Relapse treatment (n=6)	2	4	5	20
		1	3	10	30
		1	2	15	30
		1	1.8	15	27
		1	1.5	22	33
**Hepatic tumour (n=4)**	**Unspecified hepatic tumour (n=4)**				
	Primary treatment (n=1)	1	4	5	20
	Relapse treatment (n=3)	3	4	5	20
**Other and unspecified malignant**	**Pleuropulmonary blastoma (n=1)**				

**neoplasm (n=1)**					
	Primary treatment (n=1)	1	3	12	36
**Other malignant epithelial neoplasm (n=1)**	**Nasopharyngeal carcinoma (n=1)**				
	Relapse treatment (n=1)	1	4	5	20
**Total**	**167**				

n: number of courses, CNS: central nervous system, DIPG: diffuse intrinsic pontine glioma, DMG: diffuse midline glioma

Table S3. Distribution of diagnoses, number of courses, dose range and target in the group with equal or superior expected outcome (n=109)

Diagnosis	Number of courses	Range of total dose in Gy	Target
Renal tumour	45	10-25.2	Abdomen/flank (n=31), whole lung (n=14)
Neuroblastoma	17	21-30.6 Gy	Thoracoabdominal paravertebral target (n=16), femur (n=1)
Soft tissue sarcoma	16	4-56	Extremity (n=14), pelvis (n=1), neck (n=1)
Malignant bone tumour	15	15-61.2	Extremity (n=10), whole lung (n=3), pelvis (n=2)
Haematological malignancy	12	18-20 Gy	Whole brain (n=4), orbital (n=3), head/neck area (n=1), mediastinum (n=1), spleen (n=1), testicles (n=1)
CNS tumour	2	50.4-54	CNS (n=2)
Other epithelial neoplasm	2	55-66	Nasopharynx (n=1), vagina (n=1)
**Total**	**109**		

n: number of courses, CNS: central nervous system

Table S4. Distribution of diagnoses, number of courses, dose range and target in the group with uncertainty due to air/movement/metal in field (n=71)

**Diagnosis**	**Number of courses**	**Range of total dose in Gy**	**Target**
Lymphoma	32	15-34	Head/neck, mediastinum +/- axillae and/or spleen (n=32)
Neuroblastoma	11	21-30.6	Paravertebral abdominal and/or thoracic region (n=11)
Soft tissue sarcoma	11	30.6-59.4	Abdomen/pelvis (n=5), head/neck (n=4), thorax (n=2)
Malignant bone tumour	10	10-60	Thorax/vertebrae (n=6), pelvic bones (n=2), skull base (n=1), extremity (n=1)
Nasopharyngeal carcinoma	4	66-68	Nasopharynx (n=4)
Renal tumour	2	12-12.5	Abdomen (n=1), whole lung (n=1)
Ependymoma	1	50.4	Spine (n=1)
**Total**	**71**		

n: number of courses

Table S5. Distribution of diagnoses, number of courses, dose range and target in the group with favourable photon plan (n=22)

**Diagnosis**	**Number of courses**	**Range of total dose in Gy**	**Target and** ***rational for favourable photon plan***
CNS tumour	8	54-59.4	Posterior fossa (n=7). *In most cases, significant parts of the CTV were in close proximity to the brainstem, and the photon plan was assessed to have better target coverage. In one case there were concerns about elevated LET in the brainstem for a patient with preexisting central respiratory difficulties. One case was a reirradiation type 1 in a previous Gamma Knife treated area. Sharper dose gradient close to brainstem with photons.*
Soft tissue sarcoma	5	48.6-50.4	Head/neck (n=3). *VMAT-plans with lower doses to OARs and deemed more robust. One plan could spare hippocampus better. One plan was a reirradiation and due to proximity of brainstem and concerns of LET the photon plan was chosen. One plan in the epipharynx region had less dose to the visual apparatus and cochlea and was deemed more robust due to air/bone interface.*<br>Extremities (n=2). *One plan in a shallow target had a more advantageous dose distribution with tangential fields and one plan was more robust without advantages with PT.*
Lymphoma	2	28.8-30	Cervical lymph nodes (n=1). *Lower doses to parotid glands.*<br>Mediastinum (n=1). *Better target coverage with photons. More robust photon plan due to lack of motion management. The patient could not perform treatment in DIBH.*
Other epithelial neoplasm	2	50-66	Retrobulbar (n=1). *Relapse nasopharyngeal cancer. Reduced uncertainties with photons due to re-irradiation type 1.*<br>Parotid gland (n=1). *Lower doses to OAR with photons.*
Leukaemia	1	18	Relapse ALL (n=1). *Very small, superficial target in the anterior chamber of the eye. Better target coverage with photon plan with bolus, more robust.*

Malignant bone tumour	1	34.2	Whole lung and boost to rib (n=1). *Less dose to the heart with the VMAT plan.*
Neuroblastoma	1	21	Chin (n=1). *Better target coverage with photons, shallow target. More robust photon plan.*
Renal tumour	1	15	Flank (n=1). *More robust photon plan due to air and internal movement.*
Retinoblastoma	1	46	Orbitae (n=1) *Small target. Hotspots with the proton plan.*

LET=Linear energy transfer, VMAT=Volumetric Arc Therapy, OAR=Organ at risk, DIBH=Deep inspiration breath hold, n: number of courses

Table S6. Courses with a mix of photons and protons plan due to dosimetrical advantages (n=14)

**Diagnosis**	**Number of courses**	**Range of total dose in Gy**	**Target and rational for mixed dose plan**
CNS tumour	4	54-59.4	1. CSI with photons due to no possibility of anaesthesia and a more robust plan due to motion and shorter treatment time. Boost to posterior fossa with protons.<br>2. CSI with protons and boost with photons to a large volume; whole posterior fossa and a part of cerebrum.<br>3. Cerebellum. Better coverage of target with photons and lower doses to normal tissue with protons<br>4. Suprasellar region. Advantageous target coverage with photons, less dose to hippocampi with protons.<br>CSI with protons. Mixed boost to cerebrum due to lower doses to retina and optic nerves bilaterally.
Soft tissue sarcoma	4	50.4-55.8	1. Pharynx: Better target coverage with photons in the cranial part of target due to bone-air interface. Advantage with less dose to normal tissue with protons.<br>2. First whole lung with photons, then a proton boost to the maxilla<br>3. Maxilla: Better target coverage with photons due to bone-air interface. Less dose to normal tissue with protons.<br>4. Epipharynx: photon plan due to uncertainties with air. CSI with protons.

Malignant bone tumour	3	54-60	1. Whole lung with photons followed by boost to vertebrae/rib with protons<br>2. Whole lung with photons. Protons to primary tumour in pelvis, advantage due to less dose to bladder and bowel bag.<br>3. Whole lung with photons. Boost to chest wall with protons. Advantage due to less dose to normal tissue in lung.
Neuroblastoma	1	54	Primary CNS neuroblastoma. CSI with protons. Mixed boost due to possibility of lowering the dose to the visual apparatus.
Renal tumour	1	18	Whole lung with photons. Boost paravertebral with protons due to lower doses to normal tissue, healthy lung.
Retinoblastoma	1	50.4	Tissue inhomogeneities, air in the sinuses and postoperative swelling. A mixed plan was judged to be more robust.

n: number of courses, CNS: central nervous system, CSI: craniospinal irradiation
